# No association between HPV-status in tonsillar tissue and sexual behavior of the patients in a northern German population - Critical view of the link between HPV natural history and HPV-driven carcinogenesis

**DOI:** 10.1016/j.pvr.2020.100207

**Published:** 2020-09-21

**Authors:** Elgar Susanne Quabius, Asita Fazel, Christopher Knieling, Stephan Gebhardt, Martin Laudien, Crystal Moore, André Kühnel, Florian Hoppe, Robert Mlynski, Alessa Heinrichs, Alexander Fabian, Markus Hoffmann

**Affiliations:** a Department of Otorhinolaryngology, Head and Neck Surgery, Christian-Albrechts-University, Arnold-Heller-Str. 3, Building 27, D-24105, Kiel, Germany; b Environment Agency, 2 Marsham Street, London, SW1P 4DF, UK; c Department of Otorhinolaryngology, Head and Neck Surgery, Asklepios Hospital Harburg, Eißendorfer Pferdeweg 52, D-21075, Hamburg, Germany; d Department of Otorhinolaryngology-Head and Neck Surgery, Klinikum Oldenburg, Rahel-Straus-Straße 10, D-26133, Oldenburg, Germany; e Department of Otorhinolaryngology, Head and Neck Surgery, University of Rostock, Doberaner Str. 137-139, D-18057, Rostock, Germany; f Department of Radiooncology, Christian-Albrechts-University, Arnold-Heller-Str, 3 Building, D-24105, Kiel, Germany

**Keywords:** HPV, Oral, Sexual behavior, Oral sex, STD, Infection

## Abstract

HPV-infection in patients with HNSCC is reportedly correlated with sexual behavior, age, and tobacco/alcohol-consumption. HPV-infections of the oral cavity are regarded as sexually transmitted. Comparable data of patient populations outside the U.S. are sparse or missing.

Questionnaires regarding sexual behavior, education tobacco- and alcohol-consumption, were given to 28 patients with tonsillar hyperplasia (H) and 128 patients with tonsillar carcinomas (CA), all with tissue-typed HPV-DNA-status performing PCR. Answers were correlated among groups and HPV-status.

106 questionnaires were analyzed. Comparisons between H- (n = 25) and CA- (n = 81) patients showed that CA-patients were older (61.1yrs ± 9.3) than H-patients (45.2yrs ± 11.9; p < 0.0001; Student's t-test); had a lower educational level (p = 0.0095); and lower number of sexual partners (p = 0.0222; Fisher's exact test). All groups showed a significant correlation between smoking and lack of HPV-DNA-positivity (p = 0.001). Further Fisher's exact tests and logistic regression analysis revealed in all 106 patients no significant correlations between tissue-HPV-status and the analyzed parameters.

Despite the limited sample size, we were able to confirm the established correlation between smoking and tissue-HPV-status. The correlation between sexual behavior and HPV-infection was not confirmed. No consensus exists in the literature about the latter. Our data does not support the strict classification of oral HPV-infections and HPV-driven HNSCCs as STDs.

## Highlights

• Questionnaires from 81 TSCC and 25 tonsillar hyperplasia (H) patients were analyzed.
• No correlation between HPV positivity and sexual behavior was found.
• HPV positive H had both less sexual partners and oral sex than HPV negatives (trend).
• In TSCC sexual partners and oral sex were similar irrespective of HPV status.
• Incongruent data about sexual behavior, HPV transmission and cancer transgression.

## 1 Introduction

Various human cancers are causally linked to infections with human papillomaviruses (HPV). In the 1980s Harald zur Hausen and co-workers [1,2] established the carcinogenic role of HPVs in anogenital, specifically, cervix uteri cancers. He was granted the Nobel Prize for Medicine in 2008 for this work. Nowadays, it is accepted that nearly all cervix uteri cancers and roughly half of vulva, penile and anal cancers are caused by HPV [3]. Following the initial description of the presence of HPV in laryngeal papillomas [4], early studies suggested HPV as a carcinogenic agent, initiating squamous cell carcinomas of the head and neck (HNSCC) [[5], [6], [7], [8], [9], [10], [11], [12]]. Today, oropharyngeal (OSCC) and specifically tonsillar SCC (TSCC) are recognized as attributable specifically to HPV genotype 16 (HPV16) infections in 30–90% of cases, depending on the geographical region where the patients live [13]. The natural history of HPV in human mucosa and anogenital/oral HPV infections has been attributed to age at sexual debut, sexual behavior (i.e. performing oral sex) and lifetime number of sexual partners. This is based on (i) studies investigating oral and anogenital HPV prevalence and incidence in HIV-negative and HIV-positive men [among others, men having sex with men (MSM)] [[14], [15], [16]], (ii) in spouses/partners of HPV-positive people [[17], [18], [19]], and (iii) studies on vertical and horizontal HPV transmission in the general population [[20], [21], [22]]. Other socio-epidemiologic factors contributing to HPV infections are gender, age, education, income, intake of tobacco, alcohol and marijuana [23]. Existing evidence on sexual behavior and HPV incidence in anogenital lesions, benign lesions included, enables these HPV infections to be categorized as sexually transmitted, thus representing a sexually transmitted disease (STD) [24,25]. HPV transmission into the oral cavity is also discussed as being predominantly linked to sexual behavior as defined above. However, the immense evidence on natural history of HPV in the oral cavity is not fully congruent. It contradicts, at least in part, existing evidence regarding characteristics of patients with HPV driven OSCC/HNSCC. Studies regarding the natural history of oral HPV show that smokers carry a higher burden of HPV in the oral cavity compared to non-smokers [26]. It is well established that it is predominantly non-smokers who develop HPV driven OSCC [23,27,28]. HPV16 is the genotype responsible for approximately 95% of active HPV infections in OSCCs [13,28,29], however, HPV16 is rarely detected in the oral cavity in natural history studies [22,30]. The natural history of oral HPV does not seem to be directly linked to HPV-driven carcinogenesis. In recent studies of natural history data, a clear role of sexual behavior in HPV transmission is questioned [17,31,32]. Studies of HNSCCs either support [[33], [34], [35]] or contradict [35, 36 and Ref. cited therein] a predominant role of both lifetime number of sexual partners and oral sex behaviors in triggering HPV-driven carcinogenesis.

The current study in a northern German population aimed to a) investigate whether patients suffering from HPV-positive TSCCs show differences in their sexual behavior, gender, age, education, tobacco and alcohol intake when compared to HPV-negative patients; b) investigate whether HPV-positive patients suffering from tonsillar hyperplasia, specifically with obstructive sleep apnea syndrome, show differences in their sexual behavior, gender, age, education, tobacco and alcohol intake when compared to HPV-negative patients. Lastly, c) we compared the incidence of the above social parameters between the two patient groups and d) in an exploratory analysis all HPV-positive patients were compared to all HPV-negative patients, irrespective of their disease to increase our power to observe a correlation between demographic variables and the presence of HPV.

## 2 Materials and methods

### 2.1 Patient questionnaires

In a prospective, consecutive setting, patients suffering from tonsillar squamous cell carcinomas (TSCC) or obstructive sleep apnea syndrome (OSAS) due to tonsillar hyperplasia, were enrolled in this study, when treated by surgical resection at the ENT Departments of the University hospitals in Kiel and Rostock, the municipal hospital Oldenburg and at the ENT Department of the Asklepios Clinic Hamburg-Harburg. All patients had to be 18 years of age or older. Paper-based questionnaires regarding, age, gender, smoking and drinking habit, sexual orientation, lifetime number of sexual partners, frequency of oral sexual intercourse, and education were prospectively given to 28 patients with tonsillar hyperplasia (H). In addition, 128 patients with tonsillar squamous cell carcinoma (TSCC) were questioned. 53 patients in this group were enrolled in a prospective manner (pCA). A further 75 TSCC-patients with previously determined HPV-status [28] treated between 2002 and 2010 at the Department of Otorhinolaryngology, Head and Neck Surgery, CAU, Germany, were contacted retrospectively (rCA) to increase patient numbers. The applied questionnaire for analysis was based on survey instruments published and referred to by others [20]. The selection options for a) alcohol intake and b) frequency of oral sexual intercourse asked for in the questionnaires were defined for the patients as follows: a) never = no alcohol drinking at all; former = stopped drinking alcohol at least 2 years before diagnosis; rarely = 1–2 drinks per month; frequently = 1–2 drinks per week; regularly = ≥ 3 drinks per week; b) never = no oral sex at all; rarely = < 6 times a year; frequently > about once a month; regularly = more than once a month.

The study was approved by the local Ethics committee (D429/14, D534/16). Patients gave written informed consent.

### 2.2 HPV status of the tissue specimens

The HPV-DNA status in all tonsillar tissue samples was also determined in Kiel using the following experimental procedures: DNA was extracted from 4 to 6 consecutive 5 μm FFPE-sections using the ExpressArt Mag RNA + DNAready kit (AmpTec, Hamburg, Germany), according to the manufactures protocol (for the H- and pCA samples) and the QIAamp DNA Mini Kit; Qiagen, Hilden, Germany for the rCA samples. All necessary validation experiments were performed to assure comparability of the results. In all cases 50 ng of DNA per sample was used to perform PCR reactions using the primers GP5+/GP6+ as described previously [37]. DNA integrity was analyzed by performing PCR reactions using genomic beta-2 microglobulin (B2M) primers (Promolgene; Berlin, Germany) according to the manufacturer's protocol. Additionally, a positive control (a synthetic oligonucleotide of the HPV L1 gene, covered by the GP5+/GP6+ primers; Eurofins; Ebersberg Germany) was amplified in the GP5+/GP6+ PCRs. DNA-quantity and -quality were assessed using the Nanodrop 1000 (peqlab, Erlangen, Germany) and the Tapestation 2200 (Agilent, Böblingen, Germany), respectively.

All tissue samples with detectable HPV-DNA were defined as HPV-positive.

### 2.3 Statistical analysis

Firstly, demographic differences between the two cancer patient cohorts were analyzed using Fisher's exact tests and the demographic characteristics of the cancer patients of both cohorts (all CAs) and the patients with tonsillar hyperplasia (H) were compared using Fisher's exact tests. Student's t-test was performed to assess age-related differences between pCA and rCA patients and between all CA- and H- patients (all tests were performed using SPSS 20.0). The relationship between HPV positivity and the demographic variables was then assessed within each disease group and was finally assessed in an exploratory analysis in the two groups combined using Fisher's exact test. Additionally, logistic regression analysis was performed to further explore the association between the demographic variables and tissue HPV-positivity in all patients, with additional adjustment for age and smoking habit. p-values ≤0.05 were considered statistically significant, confirmed by Bonferroni post-testing for multiple comparisons where appropriate.

## 3 Results

### 3.1 Return rate

In total 28 questionnaires were prospectively given to patients with tonsillar hyperplasia with a return rate of 89% (n = 25). A total of 128 questionnaires were given to TSCC-patients. 53 patients were prospectively questioned with a return rate of 75% (n = 40). 75 TSCC-patients were retrospectively questioned to increase numbers with a return rate of 55% (n = 41). All returned questionnaires (n = 106) were analyzed irrespective of completeness. The quantity of missing data for each question is given in the respective tables.

### 3.2 Patient characteristics and comparison of the 3 cohorts

The patient characteristics of the TSCC- and H-patients are given in Table 1. Overall, there are no significant differences between the prospective and the retrospective cancer cohorts. These two cohorts were therefore pooled for further analysis. The comparison between all TSCC- (n = 81) and H-patients (n = 25) is also presented in Table 1. Patients with tonsillar hyperplasia were significantly younger than TSCC-patients [62.3 ± 9.6yrs (pCA) vs. 59.9 ± 8.9yrs (rCA), p > 0.05; 61.1 ± 9.2yrs for all TSCC vs 45.2 ± 11.9yrs for H; p < 0.0001; all data are mean ± standard deviation]. The group of TSCC-patients had significantly more former smokers than the group of H-patients (p ≤ 0.001), with an even distribution of active and never smokers in the groups of H- and CA-patients. CA-patients (n = 81) had significantly lower educational level p = 0.0095, and a significantly less promiscuous lifestyle than H-patients (p = 0.02). All other parameters, including HPV-DNA-status were not significantly different between H- and TSCC-patients.

**Table 1:** Patient characteristics of the cancer patients of the prospective (pCA), retrospective cohort (rCA), and the patients with tonsillar hyperplasia (H).

variable	cohort				Significance	
	pCA (n = 40)	rCA (n = 41)	all CA (n = 81)	H (n = 25)	pCA vs rCA	all CA vs H
Age	62.3 ± 9.6	59.9 ± 8.9	61.1 ± 9.3	45.2 ± 11.9	n.s.	p < 0.0001
sex						
female	10 (25.0)	17 (41.5)	27 (33.3)	4 (16.0)	n.s.	n.s.
male	30 (75.0)	24 (58.5)	54 (66.7)	21 (84.0)		
HPV-DNA						
negative	24 (60.0)	20 (48.8)	44 (54.3)	18 (72.0)	n.s.	n.s.
positive	16 (40.0)	21 (51.2)	37 (45.7)	7 (28.0)		
Smoking habit at time of diagnosis (n = 2 missing both pCA)						
active	13 (34.2)	13 831.7)	26 (32.9)	18 (72.0)	n.s.	p = 0.0001
never	5 (13.2)	8 (19.5)	13 (16.5)	6 (26.0)		
former*	20 (52.6)	20 (28.8)	40 (50.6)	1 (4.0)		
alcohol consumption**						
never, rarely, former	22 (55.0)	24 (58.5)	46 (56.8)	15 (60.0)	n.s.	n.s.
frequently, regularly	18 (45.0)	17 (41.5)	35 (43.2)	10 (40.0)		
number of lifetime sexual partners (n = 4 missing; 2 pCA and 2 rCA						
1–5	22 (57.9)	27 (69.2)	49 (63.6)	10 (40.0)	n.s.	p = 0.0222
≥6	16 (42.1)	12 (30.8)	28 (36.4)	15 (60.0)		
frequency of oral sexual intercourse*** (n = 9 missing; 4 pCA and 5 rCA)						
never, rarely	26 (72.2)	30 (83.3)	56 (77.8)	15 (60.0)	n.s.	n.s.
frequently, regularly	10 (27.8)	6 (16.7)	16 (22.2)	10 (40.0)		
sexual orientation (n = 17 missing; 8 pCA and 9 rCA)						
heterosexual	30 (93.8)	31 (96.9)	61 (95.3)	22 (88.0)	n.s.	n.s.
bisexual	2 (6.2)	1 (3.1)	3 (4.7)	2 (8.0)		
homosexual	0 (0.0)	0 (0.0)	0 (0.0)	1 (4.0)		
education (n = 9 missing; 3 pCA, 2 rCA and 4H)						
10yrs school****, apprenticeship	30 (81.1)	22 (56.4)	52 (68.4)	9 (42.9)	n.s.	0.0095
any university (entrance) degree	5 (13.5)	13 (33.3)	18 (23.7)	12 (57.1)		
none, other	2 (5.4)	4 (10.3)	6 (7.9)	0 (0.0)		

To define potential differences between the 3 cohorts, Fisher's exact tests were performed and the results are given as “pCA versus (vs) rCA” to analyze difference between the 2 cancer patient cohorts. The differences between the cancer patients of both cohorts (all CAs) and the prospectively analyzed patients with tonsillar hyperplasia (H) are given as “all CAs versus (vs) H”. Numbers in parentheses are percent of each cohort; *: stopped tobacco consumption at least 2 years prior to diagnosis; ** never = no alcohol drinking at all; former = stopped drinking alcohol at least 2 years before diagnosis; rarely = 1–2 drinks per month; frequently = 1–2 drinks per week; regularly = > 3 drinks per week; *** never = no oral sex at all; rarely = < 6 times a year; frequently > about once a month; regularly = more than once a month. ****: 4 years primary and 6 years secondary school; n.s.: not significant p > 0.05.

### 3.3 Effect of gender, smoking habit, lifetime number of sexual partners, frequency of oral sexual intercourse, sexual orientation, alcohol consumption and education on tonsillar HPV-DNA status

In total 37 of 81 (45.7%) TSCC-patients had HPV-DNA-positive tumors. Of the H-patients 7 of 25 (28.0%) had HPV-DNA-positive tonsillar tissues sample. In both disease entities and in the entire study population, (cancer and hyperplasia patients), patients with HPV-positive and HPV-negative tissue samples were not significantly different in age: In cancer patients with HPV-positive tumors the average age was 60.2 ± 9.4 yrs. The average age in the groups with HPV-negative tumors was 61.9 ± 9.1 yrs; Hyperplasia patients with HPV-positive tonsillar samples were on average 48.1 ± 13.4 yrs old versus 44.3 ± 11.9 yrs in the groups of patients with HPV-negative tonsillar samples. The entire population of patients with HPV-positive tonsillar tissue samples was on average 59.1 ± 10.3 yrs old and those with HPV-negative ones were on average 57.6 ± 12.3 yrs old; all p > 0.05. The effect of the social parameters on tonsillar HPV-DNA-status of the TSCC- and H- patients is shown in Table 2. None of the investigated parameters – apart from smoking habit – was associated with HPV-DNA-positivity of the tonsils. This holds true for the gender of the patients, their lifetime number of sexual partners as well as their frequency of practicing oral sexual intercourse (all p > 0.05). To further analyze the correlation between tissue HPV-positivity and the demographic variables and taking into account the limited power of our data, we performed a logistic regression analysis, followed by adjustment for age and smoking habit. The results are shown in Table 3. Due to the small sample size, logistic regression analysis could only be performed for the entire patient population (CA- and H-patients). The results presented in Table 3 corroborate the data presented in Table 2 in particular the strong association between HPV-positivity and smoking habit, which was still significant after adjusting the data for the patients' age. Interestingly, among the 25 H-patients, tonsillar HPV-negativity was associated with higher lifetime number of sexual partners. This association was found to be significant (p = 0.045; Table 2), using test for trend. Only 6 patients confirmed either homo- or bisexual orientation. No statistical analysis was possible on this small sample. Similarly, data regarding the patient's level of education was frequently missing, hindering statistical analysis. Interestingly, there is a significant correlation between smoking habit and absence of HPV-DNA-positivity of the tonsillar tissue and vice versa (p = 0.001).

**Table 2:** Effect of age, sex, lifestyle and education on tonsillar HPV-DNA status of all cancer patients (CA) and of patients with tonsillar hyperplasia (H).

variable	HPV-DNA-Status						significance (HPV-positive versus -negative)		
	positive			negative			CA only	H only	CA and H
	CA (n = 37)	H (n = 7)	CA and H (n = 44)	CA (n = 44)	H (n = 18)	CA and H (n = 62)			
Age	60.2 ± 9.4	48.1 ± 13.4	59.1 ± 10.3	61.9 ± 9.1	44.3 ± 11.9	57.6 ± 12.3	n.s.	n.s.	n.s.
sex									
female	15 (40.5)	1 (14.3)	16 (34.1)	12 (27.3)	3 (16.7)	15 (24.2)	n.s.	n.s.	n.s.
male	22 (59.5)	6 (85.7)	28 (65.9)	32 (72.7)	15 (83.3)	47 (75.8)			
smoking habit at time of diagnosis (n = 2 missing; both HPV-positive CA)									
active smoker	3 (8.6)	1 (14.3)	4 (9.5)	23 (52.3)	17 (94.4)	40 (64.5)	p = 0.001	p = 0.001	p = 0.001
never smoker	11 (31.4)	5 (74.4)	16 (38.1)	2 (4.5)	1 (5.6)	3 (4.8)			
former smoker*	21 (60.0)	1 (14.3)	22 (51.4)	19 (43.2)	0 (0.0)	19 (30.7)			
alcohol consumption									
never/rarely/former	24 (64.9)	4 (57.1)	28 (63.6)	22 (50.0)	11 (61.1)	33 (53.2)	n.s.	n.s.	n.s.
frequently/regularly	13 (35.1)	3 (42.8)	16 (36.4)	22 (50.0)	7 (38.9)	29 (46.8)			
number of lifetime sexual partners (n = 4 missing; 3 HPV-positive and 1 HPV-negative CA)									
1–5	23 (63.9)	5 (71.4)	28 (65.1)	26 (63.4)	5 (27.8)	31 (52.5)	n.s.	p = 0.045**	n.s.
≥6	13 (36.1)	2 (28.6)	15 (34.9)	15 (36.6)	13 (72.2)	28 (47.5)			
frequency of oral sexual intercourse (n = 9 missing; 8 HPV-positive and 1 HPV-negative CA) 0 (0.0)									
never/rarely	20 (69.0)	5 (71.4)	25 (69.4)	31 (72.1)	10 (55.6)	41 (67.2)	n.s.	n.s.	n.s.
frequently/regularly	9 (21.0)	2 (28.6)	11 (30.6)	12 (27.9)	8 (44.4)	20 (32.8)			
sexual orientation (n = 17 missing; 9 HPV-positive and 8 HPV-negative CA)									
heterosexual	27 (96.4)	7 (100.0)	34 (97.1)	34 (94.4)	15 (83.2)	49 (90.7)	n/a	n/a	n/a
bisexual	1 (3.6)	0 (0.0)	1 (2.9)	2 (5.6)	2 (11.2)	4 (7.4)			
homosexual	0 (0.0)	0 (0.0)	0 (0.0)	0 (0.0)	1 (5.6)	1 (1.9)			
Education (n = 9 missing; 1 HPV-positive and 4 HPV-negative CA and 4 HPV-negative H)									
10yrs school***/apprenticeship	21 (58.3)	0 (0.0)	21 (48.8)	31 (77.5)	9 (64.3)	40 (74.1)	n/a	n/a	n/a
any university (entrance) degree	9 (25.0)	7 (100.0)	16 (37.2)	9 (22.5)	5 (35.7)	14 (25.9)			
none/other	6 (16.7)	0 (0.0)	6 (14.0)	0 (0.0)	0 (0.0)	0 (0.0)			

The patients of the prospective and the retrospective cancer cohort are pooled since no significant differences between cohorts were detected (Table 1). To define potential differences between HPV-positive and HPV-negative cases in the 3 cohorts, Fisher's exact tests were performed and the results are given as “CA only” to test the correlation between the different factors and HPV-Status of the cancer patients and as “H only” to test these correlations in the group of patients with tonsillar hyperplasia. The results presented as “CA and H” relate to all HPV-negative or HPV-positive cases irrespective of their disease. Numbers in parentheses are percent of each cohort; * stopped alcohol or tobacco consumption at least 2 years prior to diagnosis; ** test for trend; *** 4 years primary and 6 years secondary school; n.s.: not significant p > 0.05; n/a: not applicable too many variables missing.

**Table 3:** Association between demographic variables and tissue HPV-positivity, all patients.

variable	univariate OR		OR adjusted for age and smoking	
	p-value	odds ratio	p-value	odds ratio
Age (years)	0.61	1.01 (0.97; 1.05)	0.52	0.99 (0.94; 1.03)
disease status				
hyperplasia (ref 1.0)				
TSCC	0.12	2.16 (0.81; 5.75)	0.06	4.00 (0.99; 17.21)
sex				
male (ref 1.0)				
female	0.18	0.56 (0.24; 1.30)	0.21	0.571 (0.24; 1.38)
smoking habit at time of diagnosis				
never (ref 1.0)				
active	<0.01	0.06 (0.02; 0.24)	<0.01	0.05 (0.01; 0.21)
former*	0.04	0.09 (0.03; 0.32)	0.06	0.08 (0.03; 0.28)
alcohol consumption**				
never, rarely, former (ref 1.0)				
frequently, regularly	0.29	1.54 (0.70; 3.39)	0.37	0.89 (0.51, 1.56)
number of lifetime sexual partners				
1-5 (ref 1.0)				
≥6	0.21	1.69 (0.75; 3.79)	0.25	1.73 (0.68; 4.43)
frequency of oral sexual intercourse***				
never, rarely, former (ref 1.0)				
frequently, regularly	0.82	1.11 (0.46; 2.70)	0.72	0.84 (0.30; 2.31)

* stopped tobacco consumption at least 2 years prior to diagnosis; ** never = no alcohol drinking at all; former = stopped drinking alcohol at least 2 years before diagnosis; rarely = 1–2 drinks per month; frequently = 1–2 drinks per week; regularly = > 3 drinks per week; *** never = no oral sex at all; rarely = < 6 times a year; frequently > about once a month; regularly = more than once a month; for education and sexual orientation too may data were missing.

## 4 Discussion

To the best of our knowledge the present study is the first prospective study with tissue typed HPV-status derived from TSCC as well as non-malignant tonsils correlating results with socioeconomic factors such as education, alcohol and tobacco intake, and sexual behavior.

The results of this study cannot confirm a correlation between the presence of HPV-DNA in the examined tissue samples (TSCC, n = 81; tonsillar hyperplasia (H), n = 25) and the sexual behavior of the patients. However, despite the relatively small number of cases studied (n = 106), the well-established and highly significant correlation between smoking habit and HPV-status was confirmed. The HPV prevalence rate of all TSCC-cases was 45.7% (27/81). This was within the expected range for this northern German study population [13,28,38].

A statistically significant relationship between lifetime number of sexual partners/frequency of oral sex and HPV-status was not observed in this study when all cases are analyzed together nor when TSCC- and H-patients were considered separately. Although power was limited, analysis for trend showed, however, that amongst the H-patients tissue HPV-negativity was associated with higher lifetime number of sexual partners (Table 2). Similar as in previous studies by us and others, here we show a significant correlation between tissue HPV-status and smoking habit of the patients (in all groups, despite limited power). Therefore, one might assume that the by others reported significant correlation between sexual behavior and tissue HPV-status should here have at least been seen in trend, also, which, however, is not the case. Due to the small number of cases altogether, one has to interpret these data with caution.

With a mean age of 45.2 years, H-patients were significantly younger than TSCC-patients (mean age: 61.1yrs), which might suggest a more liberal attitude towards sexual activity in H-patients than in older TSCC-patients. Despite the rather small H group (n = 25), it is worth noting (see Table 2) that H-patients with HPV-negative samples report a higher lifetime number of sexual partners and more frequent oral sex compared to corresponding patients with HPV-positive samples. The number of reported sexual partners and frequency of oral sex are equally divided between TSCC-patients with HPV-positive and HPV-negative tumors.

The detection of 7 HPV-positive cases among the 25 H cases was surprising. In previous studies, we have not detected HPV in clinically healthy mucosa or tonsils [[39], [40], [41]]. This discrepancy, in particular with respect to the results from non-malignant tonsils obtained by Quabius et al. [41] could be explained by the significantly younger patient population in this earlier study (mean age was 4.6 yrs) compared to the study population here with a median age of 45 years, as oral HPV prevalence has been shown to increase with age [20,42].

In the predominantly US-American literature [23,[43], [44], [45]], the correlation between sexual behavior and ano-genital/oral HPV infections [[14], [15], [16], [17], [18], [19], [20], [21], [22]] and HPV associated diseases is well established. Chaturvedi and collaborators [20], in their NHANES-based natural history study of oncogenic HPV infections in the oral cavity, suggest that the distribution of oral oncogenic HPV infections in the U.S. population is parallel to the incidence of HPV-positive oropharyngeal cancers at the population level. Infection and cancer was most common in men between 40 and 59 years who had never smoked or were former smokers. This implies a strong link between the natural history of oral HPV infection and HPV-related carcinogenesis.

However, HPV natural history studies related to the general population or MSM, and studies in HNSCC patients must be strictly separated [23,[43], [44], [45]]. Natural history studies aim to identify risk factors or markers for increased HPV prevalence/incidence, for example, in the oral cavity, based on differences in defined parameters such as gender, drug abuse, sexual behavior, etc. The key question is which parameters contribute to the transmission of HPV from person to person e.g. into the oral cavity, and who is particularly at risk. Studies in HNSCC patients have a similar design, but aim to identify factors/behaviors related to the HPV status of the tumors. The data from both study contexts allow the calculation of odds ratios for the risk of contracting (i) HPV and (ii) HNSCC. A closer examination of the results of both study types reveals correlations that require further explanation before the findings on the natural history of HPV infection can be transferred to HPV-driven carcinogenesis and vice versa: 1) The majority of studies on the natural history of HPV infection, particularly in MSM, show that anogenital and oral HPV prevalence increases with increasing lifetime number of sexual partners and with increasing number of oral sexual partners [[14], [16], [21]]. On the other hand, MSM who have recently had comparatively more oral sex partners have a significantly higher oral HPV clearance [15] than MSM with fewer oral sex partners. The majority of oral HPV infections are lost within 12 months in both MSM and the general population [14,15,17,22,46]. King and colleagues [16] have demonstrated that the overall clearance rate in MSM is higher than the incidence. 2) With few conflicting studies on natural history [47,48], smoking correlates positively with oral HPV infection and persistence of oral HPV infection [14,20,26,49,50]. In contrast, studies in cancer patients show that patients with HPV-positive OSCC are predominantly non-smokers [13,28,33,51]. 3) In 2008, Gillison and colleagues [23] published a correlation between HPV-positive HNSCC, sexual behavior and marijuana exposure. A year later, D'Souza and colleagues [48] published a significant association between open-mouth kissing and the risk of oral HPV infection. However, in a similar study, the same authors did not find an association between marijuana consumption, open-mouth kissing and oral HPV infections [52]. 4) Acquisition and persistence of oral oncogenic HPV infections are rare, even in HIV-positive MSM, and even rarer in the general population [50]. With few exceptions in natural history, oncogenic HPVs such as HPV16 are also rare among oral types [14,16,22,46]. However, HPV16 is the genotype detected in HNSCCs in over 95% of cases [13,28,29,33,51]. Unfortunately, in *in-silico* studies, the latter discrepancies are not fully taken into account when calculating odd ratios for the development of HPV16-associated HNSCC.

Syrjänen and co-workers [17,31,32] have repeatedly published results from the Finnish Family study investigating HPV transmission among spouses and siblings in a prospective manner. Results show among others that auto-inoculation from the anogenital region to the oral cavity and HPV transmission from one spouse to the other is a rare event and that HPV genotype concordance is low. Similar results have been published by others, including in three recent independent German studies [[53], [54], [55]]. All three prospective studies support the findings by Syrjänen and co-workers [17,31,32], contradicting results predominantly published for the U.S. American population [[14], [15], [16], [17], [18], [19], [20], [21], [22],^48^,^52^]. Researchers studying the U.S. American population should consider separating results from natural history studies and studies on cancer patients. This adjustment to experimental design could have a significant impact on experiments reporting that HPV acquisition and HPV-driven OSCCs are primarily due to sexual behavior. We certainly believe that sexual intercourse is a significant factor, among other parameters, affecting HPV infection rates. Risks increase with sexually riskier life-styles. On the other hand, other mechanisms of HPV transmission, apart from intense skin to skin contact, might also cause HPV-infections, as described by Sabeena and co-workers [56]. This notion is supported by data describing horizontal HPV transmission from HPV-positive mothers to their sexually inactive children [57,58] and by data describing the incidence rates of HPV infection among male virgins who did, and those who did not, initiate sexual activity during a follow-up of period of 80 months. The results were 26.2 and 16.6 cases/1000 person-months, respectively [59].

In the present study, we investigated tissue samples derived from patients, whereas all natural history and some HNSCC studies are based on DNA extracted from gargle, brushes and oral rinses respectively. Since in the head and neck region precursor lesions as described for the cervix uteri (CIN I-III) have not been detected so far and since HPV analysis on clinically healthy mucosal sites of the head and neck remain HPV negative [[39], [40], [41]], the meaning of HPV DNA detection in gargle, brushes, and rinses is not fully understood. Moreover, it has been repeatedly shown that specificity and sensitivity of the latter in terms of representing the HPV-status of the tissue samples is approximately 70% [60,61]. Therefore, results of studies relying exclusively on non-tissue samples should be interpreted with caution.

Further questions that need be addressed to fully understand the natural history of HPV and, moreover, what it takes to transgress from HPV-DNA presence in the oral cavity to HPV-driven carcinogenesis include the following: Which e.g. immunologic factors influence incident oral HPV infections to become persistent? Why do people who practice intense and regular oral sex clear HPV infections more regularly and faster than people who have less oral sex? What are the reasons for the gender-specific differences in HPV uptake and clearance? Do patients with HPV16-related HNSCCs indeed arise from the small group of individuals who have acquired a persistent HPV16 infection through riskier sexual behavior at an earlier stage of their lives, even though HPV-prevalence and HPV-associated carcinogenesis are rare events even among people at highest risk (HIV-positive MSM)? Why isn't there a significantly higher incidence of tonsillar cancers among (HIV-positive) MSM, similar to that observed for anal cancer [62]? Why do smokers in the general population have higher incidence and prevalence rates of oral HPV infections, whereas in cancer patients HPV rates are highest in the non-smoking population?

With only n = 106 investigated patients, the main limitations of the present study are sample size and the differences in age of the patient populations studied. Therefore, the pooled estimate presented should be viewed with caution and considered exploratory only. The findings within the two cohorts are likely to be less confounded but have limited power. This is the first study in Germany or even Europe on HPV-infection of tonsillar tissue of head and neck cancer patients and patients with benign lesion that also examined sexual behavior. Therefore, data presented here can only be compared to results from outside Europe. In light of detected differences in HPV prevalence rates based solely on the geographical region where patients live, comparative analysis with other study populations is challenging. We did not correlate tissue HPV DNA infection with results derived from analysis of gargle or other sample material obtained from the same patients. Therefore, comparison of our results with results obtained in biomaterial other than tissue samples is not possible. A study focusing HPV DNA status in tissue, gargle and brush samples of the same patients is ongoing.

In conclusion, in the present study no correlation between sexual behavior and HPV infection was found. The interpretation of HPV-related head and neck cancer as a STD should therefore be treated with caution. In the admittedly rather small group of H-patients, however, patients with HPV-negative tonsillar samples reported higher frequencies of practicing oral sex than patients with HPV-positive tissue samples. Overall, when communicating with patients and the public, it is important to ensure that patients with HPV-related head and neck cancer are not stigmatized. It is important to stress that HPV is widespread and HPV infections can occur in sexually active people without causing any disease. The epidemiological question of the level of risk associated with sexual activity or certain sexual activities is difficult to answer. Patient information should focus on the fact that these carcinomas have a better prognosis and not on the potential association with sexual activity. It seems important to acquire standardized high-quality risk factor data from patients originating from different geographical regions and from different study designs in order to advance our knowledge on the topic. Further studies are needed focusing on the mechanisms leading from persistent oral HPV infections to carcinogenesis.

## CRediT authorship contribution statement

**Elgar Susanne Quabius:** Conceptualization, Data curation, Formal analysis, Funding acquisition, Methodology, Supervision, Writing - original draft, Writing - review & editing. **Asita Fazel:** Conceptualization, Data curation, Methodology, Validation. **Christopher Knieling:** Investigation. **Stephan Gebhardt:** Data curation, Investigation. **Martin Laudien:** Formal analysis, Methodology, Validation. **Crystal Moore:** Writing - review & editing. **André Kühnel:** Data curation, Investigation, Supervision. **Florian Hoppe:** Data curation, Investigation, Supervision. **Robert Mlynski:** Data curation, Supervision. **Alessa Heinrichs:** Investigation. **Alexander Fabian:** Data curation, Investigation. **Markus Hoffmann:** Conceptualization, Formal analysis, Methodology, Project administration, Supervision, Writing - original draft, Writing - review & editing.

## Declaration of competing interest

The authors declare the following financial interests/personal relationships which may be considered as potential competing interests: The work was funded by the 10.13039/501100005972Deutsche Krebshilfe, grant number 111777.
